# Resilience to hydrological droughts in the northern Murray-Darling Basin, Australia

**DOI:** 10.1098/rsta.2021.0296

**Published:** 2022-12-12

**Authors:** R. Quentin Grafton, Long Chu, Richard T. Kingsford, Gilad Bino, John Williams

**Affiliations:** ^1^ Crawford School of Public Policy, The Australian National University, Canberra, Australian Capital Territory 2601, Australia; ^2^ Centre for Ecosystem Science, School of Biological, Earth and Environmental Sciences, University of New South Wales, Sydney, New South Wales 2052, Australia

**Keywords:** Budyko, resistance, recovery time, climate change, anthropogenic drought, drought resilience

## Abstract

We respond to the problem of declining streamflows in the northern Murray–Darling Basin, Australia, a region that suffers from hydrological droughts and a drying trend. We partitioned the effect of meteorological trends from anthropogenic drivers on annual streamflow, quantified the effect of annual streamflow decline on waterbird abundance, estimated the effects of streamflow change on a measure of ecosystem resilience, and calculated the net benefits of in-stream water reallocation. The anthropogenic drivers of hydrological droughts were assessed by comparing the Lower Darling (hereafter the Barka) River, which has large recorded water extractions, with the adjacent Paroo River, which has very little recorded water extractions. Findings include: (1) only about one-third of the recent reduced streamflow of the Barka River is due to a meteorological drying trend; (2) statistically significant declines in waterbird species richness and abundance have occurred on both rivers between 1983–2000 and 2001–2020; (3) declines in waterbird abundance have been much larger along the Barka River than the Paroo River; and (4) ecosystem resilience, as measured by waterbird abundance, wasgreater on the Paroo River. Our four-step framework is applicable in any catchment with adequate time-series data and supports adaptive responses to hydrological droughts.

This article is part of the Royal Society Science+ meeting issue ‘Drought risk in the Anthropocene’.

## 1. Introduction

Anthropogenic water extractions tripled over the period 1960–2010 [1], with further increases projected [1–3]. These extractions have contributed to the degrading of many of the world's rivers and associated ecosystems [4,5]. A projected drying trend in all habitable continents [6–11] may at least double the frequency of extreme droughts in some regions [12]. The largest drying impacts are projected to occur in the mid-high latitudes [13] and will further exacerbate riparian declines in the absence of good understanding and effective, evidence-based adaptive responses. Identifying what the adaptive responses should be in relation to declines in streamflow requires, at a minimum, answers to three critical questions: (1) What is the proportion of observed declines in streamflow attributable to long-term meteorological trends versus direct anthropogenic drivers? (2) What are the impacts of reduced streamflow on ecosystem resilience? (3) What are the costs and benefits of in-stream water reallocation in response to streamflow and ecosystem decline?

We respond to these three critical questions and focus on *drought resilience* actions [14] intended to support social–ecological systems to ‘…anticipate, absorb, accommodate or recover from the effects of drought in a timely and efficient manner, including through ensuring the preservation, restoration, or improvement of natural capital’ [15, p. 2] and resilience-based approaches to climate change [16,17]. Drought resilience is a response to *anthropogenic drought*, which includes the full spectrum of processes within human–nature systems [18]. In particular, anthropogenic drought is influenced by social–ecological–economic drivers affecting hydrological systems [19] and can be cumulative and additive to ‘natural causes’ of drought. Anthropogenic drought encompasses: (1) *meteorological drought*, arising from a lack of precipitation over a region for a period of time [20]; (2) *hydrological drought*, arising from insufficient river streamflow and/or water storage in reservoirs, lakes and groundwater aquifers that are below long-term mean levels [21]; and (3) *agricultural drought*, arising from soil moisture deficits and insufficient water for crops, pasture and livestock production [20,21].

**Figure 1.**
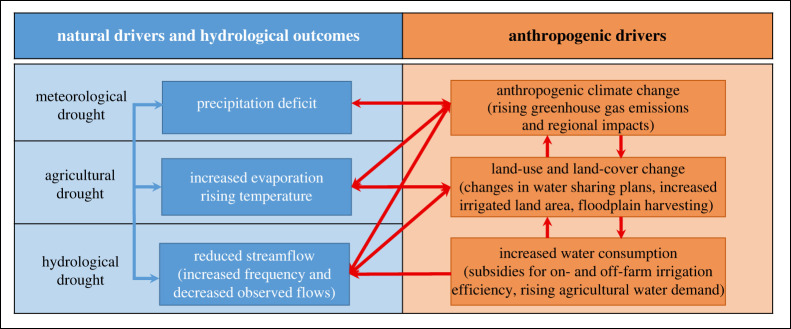
Anthropogenic drought and the Barka River, Australia. Adapted from [18]. The text in the boxes and the arrows are provided by the authors and are specific to the Barka River. (Online version in colour.)

Figure 1 illustrates the natural drivers and anthropogenic drivers, and their interactions, in relation to anthropogenic drought for the Lower Darling River, known as the Barka to the Indigenous people of the river, the Barkandji Nation. The Barka River is located within the northern Murray–Darling Basin (MDB), Australia. Figure 1 highlights that anthropogenic climate change is a global-scale phenomenon that is contributing to a precipitation deficit in the Barka catchment and meteorological drought. At a regional scale, land-use change, such as increased water extractions for irrigation and the choice of irrigated crops [20] along the Barka River, have contributed to increased evaporation. These direct human influences, along with a ‘natural’ trend of increased temperatures, have contributed to agricultural drought in the catchment. Hydrological drought is affected by both meteorological factors (e.g. a drying trend which may be influenced by anthropogenic climate change) and direct human influences [22] at a regional scale, such as water extractions, storage of water from floodplains [23] and irrigation efficiency [24].

We studied the causes, consequences and possible adaptive responses to hydrological drought in the northern MDB of southeastern Australia. Our findings are of general interest because: (1) this basin is one of the most variable regions of the world in terms of streamflow and precipitation [24] and is likely to become drier with ongoing climate change [25]; (2) the basin is characterized by very large water extractions as a proportion of estimated inflows [5], especially during droughts [26]; (3) the basin is subject to ‘fragile water security’ and hydrological complexity [27], notwithstanding very large public investments in water infrastructure and government efforts to reallocate water among competing uses [28]; and (4) Australian water governance has been highlighted in international comparisons [29].

Our study applied four analytical steps that we developed, and that are replicable in catchments elsewhere in the world, to analyse hydrological drought and opportunities to improve drought resilience. These steps include: (1) partitioning long-term meteorological trends from anthropogenic drivers of hydrological drought such as land-use change or changes in water consumption; (2) quantifying the effects of hydrological drought on ecosystems services; (3) evaluating ecosystem resilience, or the ability to ‘bounce back’ [30], following a hydrological drought; and (4) estimating the possible costs and benefits of water reallocation to increase streamflow as an adaptive response to mitigate decline in ecosystem resilience from hydrological droughts.

For the northern MDB, we analysed: (1) meteorological trends (air temperature, precipitation, evaporation, seasonality of precipitation) that, in part, are influenced by climate change; (2) trends in streamflow on the Barka River and the Paroo River partitioned by meteorological trends and direct human influences; (3) waterbird abundance at two wetlands—Menindee Lakes, adjacent to the Barka River, and the Paroo River Wetlands, a wetland designated under the Ramsar Convention—and the relationships between waterbird abundance and streamflow; and (4) the resilience of waterbird abundance, as measured by resistance and recovery time, under alternative water reallocations and the associated economic trade-offs for the Barka River.

## 2. Northern Murray–Darling Basin

The MDB encompasses a large area of southeastern Australia exceeding 1 million km^2^. It is home to about 2.2 million people and more than 30 000 wetlands—16 of international importance under the Ramsar Convention [31]. The two main rivers in the MDB are the Darling River and the River Murray. The Darling River begins with tributaries in northeastern New South Wales (NSW) and southern Queensland, and then flows south through western NSW to join the River Murray at the border of NSW and the state of Victoria.

Key recent water reforms, relevant to our analyses, over the past two decades [32] include: (1) the 2004 National Water Initiative, an intergovernmental agreement on principles, and associated actions, in relation to water governance agreed to by the federal government and state governments; and (2) the Water Act 2007 that gave the Australian federal government powers in relation to water planning in the MDB to ensure sustainable levels of water extractions. The 2012 MDB Plan gives effect to the Water Act 2007 and established basin-wide and catchment-level sustainable diversion (extraction) limits (SDLs) for both surface water and groundwater [33,34]. These SDLs are intended to give effect to key objects of the Water Act 2007 including 3d(i) ‘…the return to environmentally sustainable levels of extraction for water resources that are overallocated or overused’ and 3d(ii) ‘…to protect, restore and provide for the ecological values and ecosystem services of the Murray–Darling Basin’.

**Figure 2.**
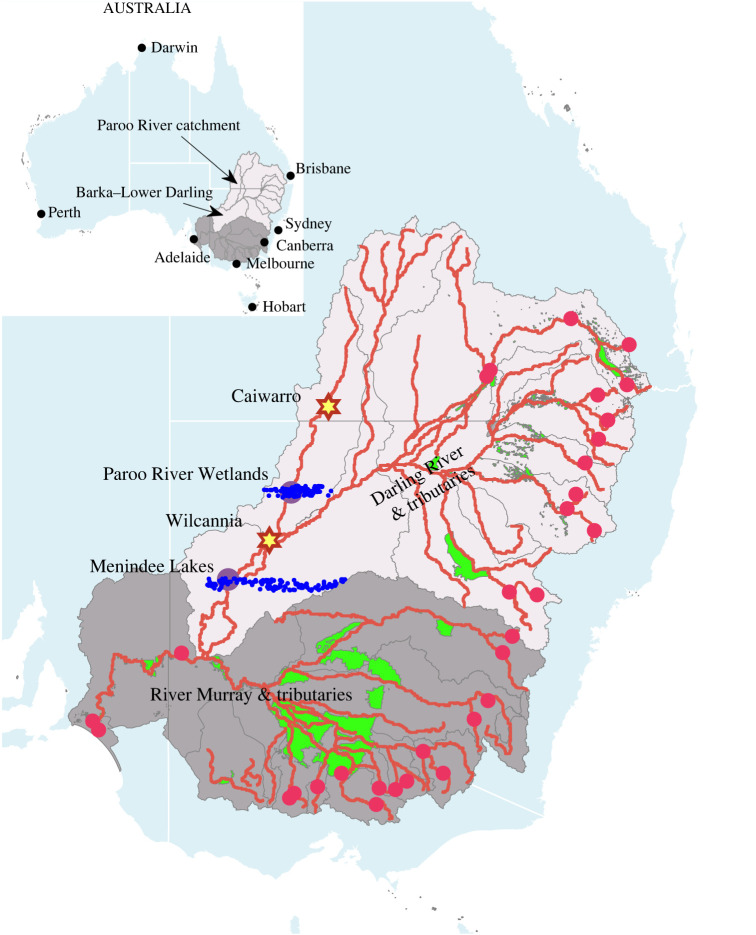
Murray–Darling Basin and study locations. The northern MDB is denoted by the off-white (chalk) area. The southern MDB is denoted by the dark-grey area. Red (largest) dots represent major water storages. Red lines are main river channels. Grey lines in the MDB region are boundaries of catchments. Green shaded areas are indicative of irrigation districts. Stars are streamflow gauging stations. Purple dots are study locations. Blue (smallest) dots are the aerial survey locations for waterbirds. Not all locations were surveyed in all years. Data sources: BoM, MDBA and Kingsford *et al*. [41] (see details in appendix A).

Our analyses focused on two adjacent catchments and two wetlands within the northern MDB, both located in northwestern NSW. The first wetland is at Menindee Lakes, NSW, adjacent to the Barka River. The Barka River, NSW, is subject to large water extractions in its upstream tributaries, declining streamflow [35] and periodic hydrological droughts with large ecological [36,37] and economic impacts, including massive fish kills [38,39] and degraded ecosystem health [40]. The second location is the Paroo River Wetlands, NSW, northwest of the Barka River (figure 2), with streamflow supplied by the largely free-flowing Paroo River, which has virtually no recorded water extractions [42,43].

### (a) The Barka River, Menindee Lakes and associated wetlands

The Barka catchment has an area of about 650 000 km^2^ and receives water from the Condamine–Balonne, Macintyre, Gwydir, Namoi, Castlereagh and Macquarie tributaries, which drain the higher-rainfall, western margins of the Great Dividing Range in northern NSW and southern Queensland (figure 2). By contrast, the Paroo River has its headwaters in the more arid west and is only an intermittent contributor, during intense wet periods, to flows in the Barka River [44,45]. The six northeastern tributaries of the Barka River include nine major headwater dams (see major storages identified in figure 2) with a combined storage capacity of 4415 GL. Streamflow regulation of these upstream tributaries is high [45]; there are some 15 main channel weirs and over 1000 small weirs that cover about 1000 km (or 40% of the length) of the entire Darling River [46,47] and its streams and anabranch channels. This water infrastructure is in addition to many off-river storages on the floodplain where unregulated river flows can be extracted [38,48] for irrigation.

Large water extractions for irrigation along the Darling River, primarily in its upper catchment and its tributaries, contributed to the 1991 blue–green algal bloom that stretched for over 1000 km [49] and also to declines in abundance and diversity of native fish [50]. The possible effects of water extractions on streamflow were investigated following the 2019 Menindee fish kill [51]. This investigation highlighted the importance of habitat connectivity for fish spawning and fish movement along the Darling River, including its lower reaches known as the Barka River [38]. By comparison, the Paroo (and Warrego) Rivers are the only major catchments in the northern MDB without large dams or large water extractions, and there is little or no regulation of their natural streamflow [43].

The Menindee Lakes are a cluster of neighbouring natural deflation basins of the Barka River (54), ranging from 103 to 15 900 ha in size, that intermittently fill following flood events and are located at Menindee. The wetlands associated with these lakes include billabongs, channel complexes, backwaters, riverine benches, saline lakes, lignum swamps, deep riverine pools and extensive floodplains. The Menindee Lakes were transformed into 12 storage lakes from 1960 with the construction of four weirs, 11 regulators, seven block banks and about 15 km of constructed channels and levee banks [52]. As a result of these constructed storages, water is stored perennially in some of these lakes [53]. Notably, water collected by Lake Wetherell (a dam across the Barka River) is stored in Lakes Pamamaroo, Menindee and Cawndilla as well as smaller storage lakes. Stored water from Menindee Lakes is released directly into the Barka River either through outlets on the main weir or via Lakes Wetherell, Pamamaroo and Menindee. Water can also be released from Lake Cawndilla, via a constructed channel for downstream irrigation or to supply the Barka River Anabranch and the Barka River and the River Murray [54].

### (b) The Paroo River and associated wetlands

The Paroo River and Cuttaburra Creek are the major streams which supply the Paroo River Wetlands, a Ramsar Wetland of International Importance (figure 2). The Paroo River Wetlands include large lakes, tree-lined creeks and waterholes, lignum, cane grass swamps and artesian mound springs. The Wetlands encompass a Ramsar site containing one of the last remaining unregulated wetland systems in NSW.

The Paroo River Wetlands contain several threatened plant and animal species, migratory bird species and significant native fish communities, and are of important cultural value for Indigenous peoples ([55], pp. 34–38; [56], pp. 12–18). Notably, the Paroo River was also the only catchment in the MDB to consistently receive a ‘good’ rating in terms of ecological health, fish, macro-invertebrates, vegetation, physical form and hydrology in the Sustainable Rivers Audits [40,57]. Importantly, the Paroo River is unique in that its bed and floodplain dynamics are largely unmodified from reference conditions, as determined by the second Sustainable Rivers Audit [40]. Two features of the Paroo River Wetlands are of critical importance for waterbird conservation: the first is lignum, which is the main component of waterbird nesting habitat [58], and the second consists of the flooding events [43].

## 3 Material and methods

### (a) Data sources

We collated meteorological and hydrological data from gauging stations, previously published modelling data and indicators published by Australia's Bureau of Meteorology (BoM), the Murray–Darling Basin Authority (MDBA) and the government of Queensland (SILO). Our study also used aerial waterbird survey data that only began in 1983 and has been collected annually every year since the work of [41] within 30 km-wide survey bands across the Paroo River Wetlands, Menindee Lakes and adjacent areas (figure 2). The abundance of waterbirds was proxied by aggregating the waterbird count in a year across survey locations within each of the two study locations (i.e. Paroo River Wetlands and Menindee Lakes) and adjacent areas. Given that the annual waterbird surveys occur every October, we defined the corresponding water year as the 12 months from the previous October to September and then calculated annual indicators within a water year, unless otherwise indicated. All data are accessible from the authors, and full details of the specific data sources by table and figure are provided in appendix A.

### (b) Streamflow

As step one of our four-step analyses, we first estimated the impacts of long-term meteorological trends on streamflow using a method [59,60] based on the Budyko framework [61]. This method partitions long-term changes in water resources [62] into anthropological–hydrological impacts of land use [63,64] and meteorological trends [60,65–67]. The purpose of the Budyko analysis is to partition the effects of long-term meteorological trends from anthropogenic drivers of changes in streamflow. This method is attributed to Budyko [61] and has been widely applied in multiple catchments in Australia, India, China and the United States [59,60,62,65–67]. The method assumes that the relationship between evapotranspiration and precipitation in a catchment depends on the long-term climatic dryness index (i.e. the ratio of demand for evaporation to precipitation).

The Budyko approach is formalized in equation (3.1), where *E*_tran_ is evapotranspiration, *P* is precipitation, *E*_d_ is the demand for evaporation and *ω* is a parameter or a vector of catchment-specific parameters:

3.1 $$\frac{E_{\text{tran}}}{P}=g\left(\frac{E_{\text{d}}}{P}\;|\;\omega \right),$$

Various functional forms have been proposed for the Budyko analysis with different interpretations and values of parameters ([59], p. 6]). Here, we use Fu's function for the Budyko analysis [68,69] in equation (3.2), where *ω* > 1:

3.2 $$g\left(\frac{E_{\text{d}}}{P}\;|\;\omega \right)=1+\frac{E_{\text{d}}}{P}-{\left(1+{\left(\frac{E_{\text{d}}}{P}\right)}^{\omega }\right)}^{1/\omega }.$$

Estimation of (3.2) requires that the streamflow out of a catchment be defined as the residual function of rainfall and evapotranspiration, i.e. $f(P,E_{\text{d}}|\omega )\equiv P-E_{\text{trans}}=P-P\times g(E_{\text{d}}/P|\omega )$. We denote by $\epsilon_{f,x}{|}_{\omega }\equiv (\partial f/\partial x){|}_{\omega }(x/f)$ the elasticity of the function *f* with respect to the variable *x* ∈ [*P*, *E*_d_] and obtain equation (3.3), where the left-hand side is the percentage change in long-term streamflow and the right-hand side includes the percentage changes in long-term precipitation and maximum evaporation, weighted by the elasticity of the streamflow with respect to each variable:

3.3 $$\frac{\text{d}f}{f}{|}_{\omega }=\epsilon_{f,P}{|}_{\omega }\frac{\text{d}P}{P}+\epsilon_{f,E_{\text{d}}}{|}_{\omega }\frac{\text{d}E_{\text{d}}}{E_{\text{d}}}.$$

Equation (3.3) was used to estimate the parameter *ω* with catchment-level information about the long-term percentage changes of streamflow, precipitation and maximum evaporation. Given that the collection of class-A pan evaporation data only began in the 1970s in most gauging stations in the MDB, we focused our analysis on the post-1980 period, i.e. the 40 years from 1981 to 2020, when high-quality data were available. To estimate the long-term percentage change of each indicator, we calculated, as our base case, the average of the indicator over the first 20 years of the 40-year period, from 1981 to 2000, and compared it with the average over the most recent 20 years, from 2001 to 2020.

To estimate the parameter *ω*, we used data from the Paroo River, which has had very little recorded water extractions such that any long-term trend in streamflow is attributable exclusively to long-term meteorologically related factors. Following Roderick & Farquhar [60], the demand for evaporation was estimated by *E*_d_ = *k* × *E*_pan_ where *E*_pan_ is pan evaporation and the baseline value for *k* was 0.75. We estimated the value for parameter *ω* that matched equation (3.3) for the data of the Paroo River and used this parameter estimate to calculate the possible long-term meteorological effects on streamflow in the two catchments (Paroo River and Barka River). We also undertook sensitivity analysis by varying the parameters *k* and *ω* and investigated the robustness of our estimates. Further, we compared our estimates of long-term meteorological trends in streamflow with the hydrological modelling [70] of the MDBA, the federal agency responsible for the delivery of the 2012 MDB Plan. To do this we chose the baseline value of *k* = 0.75 and used the estimated baseline value of *ω *≈ 1.4 and distributions constructed with 10^6^ simulations.

### (c) Waterbirds

As step two of our four-step analyses, we investigated the relationship between changes in streamflow and waterbird abundance. While multiple factors influence waterbirds, our time-series analyses focused on the association between waterbird abundance and annual streamflow, noting that there is good evidence [71–73] that the distribution and abundance of different waterbird communities are related to the availability of streamflows and flooding. Given that the aerial waterbird surveys spanned only 38 years (1983 to 2020), we avoided overfitting our models by combining reduced forms with the selection criteria of time-series statistical models. Thus, our estimated models did not include all the possible causal factors that may affect waterbird abundance [73–75], but we contend that many of these influencing factors are associated with annual streamflow [76,77].

Waterbird abundance in region *r* ∈ {Paroo, Menindee} at time *t* is specified by equation (3.4). This equation was formalized in a reduced form to ensure there was no endogeneity bias. The left-hand side variable (Bird*_r_*_,*t*_) is the endogenous waterbird abundance, and all variables on the right-hand side were treated as exogenous. Specifically, *H*(*x_t_*) is the historical information set of variables *x* before time *t*; Flow*_r_*_,*t*_ is the streamflow at location *r* at time *t*; Flow_o__,*t*_, the overall streamflow measure of the entire MDB, was included to capture possible inter-regional impacts.

3.4 $$E(\text{Bir}{\text{d}}_{r,t})=f_{r}(H(\text{Bir}{\text{d}}_{r,t}),\text{Flo}{\text{w}}_{r,t},H(\text{Flo}{\text{w}}_{r,t}),\text{Flo}{\text{w}}_{o,t},H(\text{Flo}{\text{w}}_{o,t})).$$

We linearized equation (3.4) as an autoregressive distributed lag (ADL) specification [78] and obtained equation (3.5). There are three groups of independent variables in equation (3.5). The first group on the right-hand side includes the lag(s) of waterbird abundance used to capture historical dynamics. The second group represents current and historical localized streamflow. The third group is the streamflow in the entire MDB, to capture possible inter-regional bird migration impacts. In equation (3.5), *B*_r_, *F*_r_ and *F*_o_ are the lag lengths of the ADL specification, noting that we investigated several combinations of the lag lengths in our statistical analyses.

3.5 $$E(\text{Bir}{\text{d}}_{r,t})=\beta_{r,0}+\overset{B_{r}}{\underset{j=1}{\sum }}\gamma_{r,j}\text{Bir}{\text{d}}_{r,t-j}+\overset{F_{r}}{\underset{j=0}{\sum }}\alpha_{r,j}\text{Flo}{\text{w}}_{r,t-j}+\overset{F_{\text{o}}}{\underset{j=0}{\sum }}\gamma_{r,j}\text{Flo}{\text{w}}_{\text{o},t-j}.$$

Given the relatively small sample size and the fact that the degrees of freedom decline in the lag lengths, we limited the combinations of the model specifications to *B_r_*, *F_r_*, *F*_o_ ≤ 2. We chose the (set of) appropriate lag lengths using the Bayesian information criterion (BIC) as selection criterion. As the waterbird counts can be zero in some years, i.e. the wetland was dry at the survey time, a logarithm specification for analysis in abundance trends was not applied because of zero observations. Instead, we used level–level and Poisson regressions to retain all possible observations in our estimated models (see appendix B).

### (d) Ecosystem resilience

Step three of the four-step analyses evaluated the riparian ecosystem of the two rivers, as proxied by waterbird abundance, using the resilience criteria of *resistance* and *recovery time*, both of which are well developed and defined in the literature [30]. Resistance measures the proportional decline in waterbird abundance from the commencement of a drought, while recovery time is the time interval for waterbird abundance to recover to a neighbourhood of pre-drought levels. We evaluated both measures of resilience with respect to waterbird abundance in response to three different hydrological drought scenarios.

To produce numerical estimates of resilience, our simulations assumed that waterbird abundance remained at its median level (referred to as the long-term median level) *before* a modelled drought. Our simulations were such that when a modelled drought began, streamflow declined to the first quartile of annual local streamflow for a given number of years, referred to as the drought length. To consistently compare across different scenarios, we assumed that after a modelled drought ended, streamflow recovered to allow waterbird abundance to return to its long-term median level.

### (e) Trade-offs of water reallocation

The fourth and final step of our analyses estimated the possible economic losses of in-stream water reallocations to reduce upstream water extractions and, thus, increase streamflow for the Barka River. These losses were calculated as the reduction in irrigation benefits because of lower upstream water extractions. For this estimation, we combined the price elasticity of irrigation water estimated by Wheeler *et al*. [79], the observed and relevant water allocation prices in the MDB ([80], f3), and the extractions in the three most-recent dry years, i.e. 2012–13, 2013–14 and 2018–19 [81], to calibrate the demand function for irrigation water.

**Figure 3.**
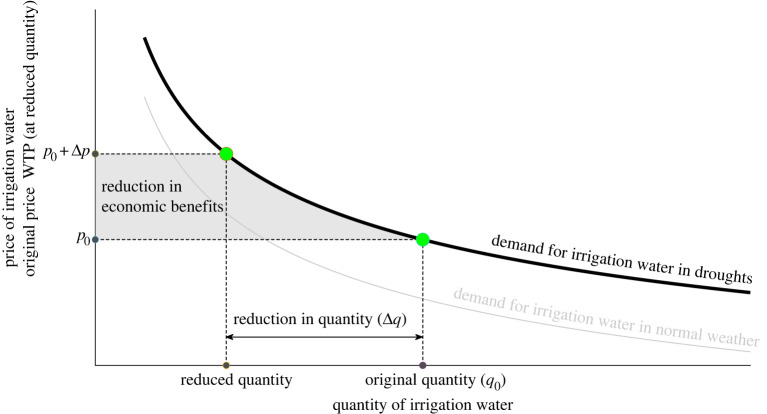
Irrigation demand for water extractions. Source: the authors. (Online version in colour.)

The response of economic benefit to changes in irrigation quantity is depicted in figure 3. In this figure, the estimated change in profits from irrigation as a result of a water reallocation is measured by the area below the demand curve of irrigation water between the original price and the willingness to pay (WTP) at the reduced quantity following a water reallocation to increase streamflow. This change is formalized in equation (3.6), where *q*_0_ and Δ*q* are the quantity and the reduction in irrigation water quantity, *h*(*p*) is the demand function and *h*^−1^ is the inverse demand function:

3.6 $$\Delta \Pi =\int_{h^{-1\;}(q_{0})}^{h^{-1}(q_{0}-\Delta q)}h(p)\;\text{d}p\;\text{.}$$

## 4. Results

### (a) Meteorological time trends

**Figure 4.**
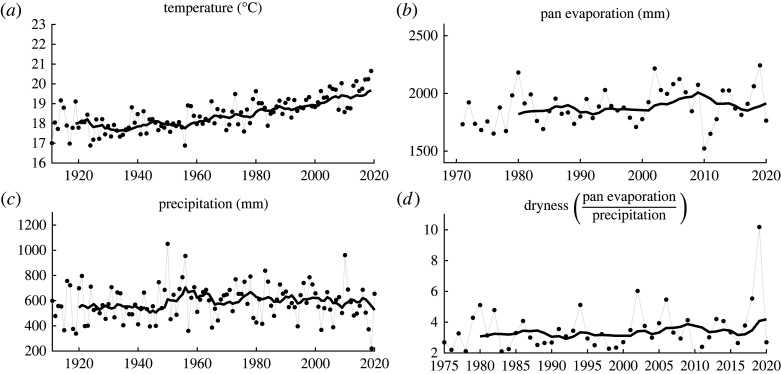
Meteorological indicators (*a*) temperature, (*b*) precipitation, (*c*) pan evaporation and (*d*) dryness in the northern MDB with solid lines representing 10-year moving averages.

Annual meteorological metrics (temperature, precipitation and pan evaporation) varied substantially across the northern MDB (figure 4). Average temperature significantly increased in the northern MDB (figure 4*a*), on average, by 0.18^°^C per decade (95% CI: +0.17 to +0.19^°^C) over the twentieth century, but accelerated over the past 40 years (1981 to 2020) to 0.26^°^C per decade (95% CI: +0.023 to +0.029^°^C). Annual precipitation was highly variable (figure 4*b*), with an overall increase over the twentieth century of 5 mm per decade (95 %CI: +2.8 to +8.0 mm), but with a negative trend over the past 40 years (−11 mm per decade, 95 %CI: −16.7 to −4.8 mm). Pan evaporation (figure 4*c*) increased since 1970 when reliable data were first collected (class-A pan evaporation), with an average increase of 21 mm per decade (95% CI: +11.1 to +31.7 mm). The decline in precipitation and the increase in pan evaporation combined, over the past 40 years, has resulted in an increase in the dryness indicator (figure 4*d*), with an average increase of 0.14 per decade (95% CI: +0.07 to +0.20). All 10-year moving average trends of the meteorological indicators in figure 4 were statistically significant (*p *< 0.001).

### (b) Streamflow trends

**Figure 5.**
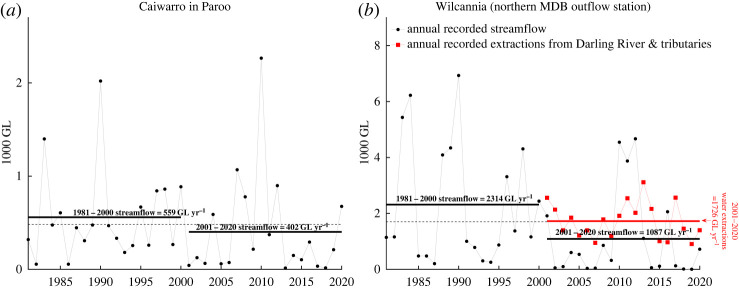
Recorded water extractions (Barka River and its tributaries) and observed annual streamflow at Caiwarro (Paroo River) and Wilcannia (Barka River), 1981–2020. Streamflow data were obtained from the BoM (Caiwarro: station #424201A; Wilcannia: station #425008). Annual water extraction data were obtained from MDBA's annual Water Audit Reports (1997–98 to 2009–10) and Cap Register 2019–2020 and NSW's General Purpose Water Accounting Reports (2010–11 to 2018–19). Horizontal dashed lines are the 40-year averages and horizontal black lines are the averages of the first and second 20 years. For visual comparison of relative changes, the two panels are scaled to align their 40-year average levels. The annual average of observed streamflow at Caiwarro (Paroo River) in panel (*a*) was 480 GL per year, 559 GL per year and 402 GL per year for the entire 40-year period (1981–2020), the first 20 years (1981–2000) and the second 20 years (2001–2020), respectively. The annual average of observed streamflow at Wilcannia (Barka River) in panel (*b*) was 1700 GL per year, 2314 GL per year and 1087 GL per year for the entire 40-year period (1981–2020), the first 20 years (1981–2000) and the second 20 years (2001–2020), respectively. The time trend in panel (*a*) is −5.2 GL per year with *p *= 0.46, and the time trend in panel (*b*) is −51.3 GL per year with *p < *0.05. Annual recorded extractions for the Paroo River were not included in panel (*a*) as their average is less than 0.5 GL per year with the highest value being 4 GL per year. (Online version in colour.)

For both the Paroo River and the Barka River, there has been a long-term decline in annual streamflow over the past 40 years. For the Paroo River, as measured at the gauge station of Caiwarro near the NSW–Queensland border, the average streamflow over the period 1981–2000 was 559 GL per year, while from 2001–2020 it averaged 402 GL per year (figure 5*a*), a decline of 157 GL per year, or 28%. For the Barka River, as measured at the gauge station at Wilcannia, average streamflow over the period 1981–2000 was 2314 GL per year, while from 2001–2020 it averaged 1087 GL per year (figure 5*b*), a decline of 1227 GL per year, or 53%. The recorded annual water extractions for the Barka River, and upstream, are from river offtakes and almost entirely for irrigation. For the catchments upstream of Wilcannia from 2001, recorded extractions were highly variable and averaged about 1726 GL per year after 2000. In periods of low inflows, during droughts, these levels of water extractions can represent 70–80% of the annual streamflow [26,37,45,82].

### (c) Budyko analyses and drivers of streamflow declines in the northern MDB

**Figure 6.**
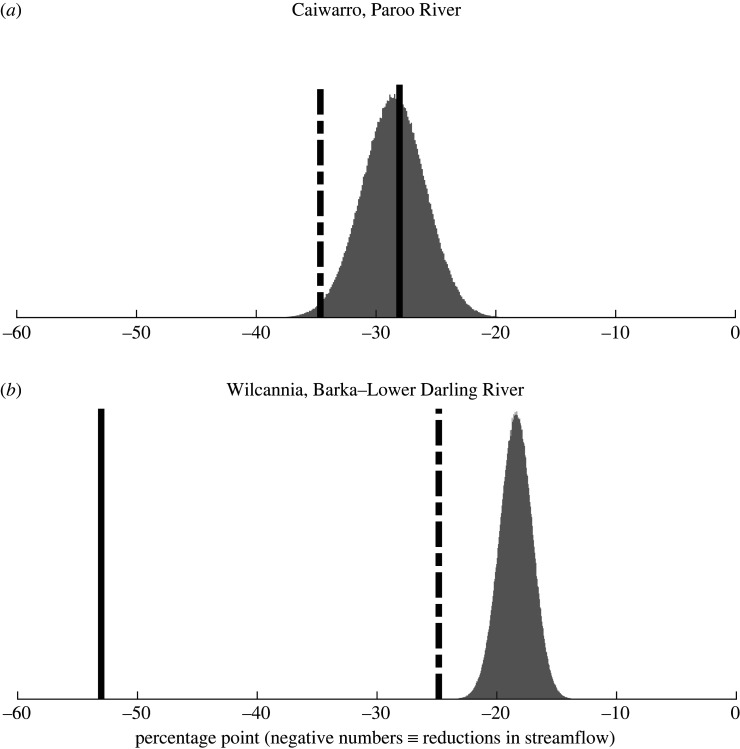
Estimated long-term meteorological trends and measured decline in streamflow (baseline case: equal time-length delineation): (*a*) Caiwarro, Paroo River; (*b*) Wilcannia, Barka–Lower Darling River. Trends were defined by changes in average streamflow in the second 20-year period (2001–2020) compared to the first 20-year period (1981–2000). Grey distributions are the long-term meteorological trends estimated using the Budyko method. Dashed lines are the climatic impacts estimated by MDBA hydrological modelling (missing numbers were interpolated by nearest-neighbour regressions in relation to rainfall, pan evaporation and months). Solid lines are the observed reductions in streamflow (see also figure 5).

Our Budyko analyses are summarized in figure 6. Measured streamflow in the Paroo River catchment declined by 28% from the 1981–2000 period to the 2001–2020 period ( solid vertical line, figure 6*a*). The Budyko estimates indicated a possible range of long-term meteorological impacts on streamflow of between 17% and 41%, with a 99% confidence interval of  [22%, 35%] (the distribution in figure 6*a*) and a mean decline of 28%. Separate hydrological modelling undertaken by the MBDA [70] projected that a drier climate would have reduced the streamflow on the Paroo River by 35% (dashed line in figure 6*a*).

For the Barka River at Wilcannia, the Budyko analysis indicated that the streamflow reduction caused by the drying trend over the past 40 years was between 12% and 27% (99% CI: 15% to 25%) (the distribution in figure 6*b*) with a mean decline of 18%. This range encompassed the projected reduction by the MBDA [70] of around 25% (dashed line in figure 6*b*) associated with a drier climate. By comparison, the observed reduction of 53% (the solid line in figure 6*b*) in streamflow on the Barka River at Wilcannia was much larger than predicted by the Budyko analysis. This difference between the observed and the lower predicted reduction in streamflow from the Budyko analysis indicates that climate change alone does not explain the observed reduction in streamflow over the past 40 years. Instead, our analysis indicated that both climate change and direct human influences [22], such as from land-use change and water extractions, are responsible for the decline in streamflow on the Barka River.

We undertook a sensitivity analysis of the time-length delineation associated with changes in streamflow over the past 40 years, and these results are summarized in table 1. The long-term meteorological trend streamflow reduction estimates are reported as a mean and a range (in square brackets) for each case. At Wilcannia, when using the year 1996 (the year after a cap on MDB surface-water extractions was introduced) as the breakpoint for the time-length, the estimated long-term meteorological trend of streamflow reductions ranged from 3.6% to 20.2% with a mean of 11.5% (95% CI: 8.1% to 15.0%). The overall observed streamflow reduction between the first (1981–1995) and second (1996–2020) periods was 38.9%. When using the year 2005 as the breakpoint for the time-length, the estimated long-term meteorological trend ranges from 8.4% to 11.8% with a mean of 10.1% (95% CI: 9.4% to 10.8%). The overall observed reduction between the first (1981–2005) and second (2006–2020) periods was 37.5%.

**Table 1.** Time-length sensitivity analysis of long-term meteorological influence on observed streamflow.

	Caiwarro, Paroo River		Wilcannia, Barka River	
Unit: %	long-term meteorological trend streamflow reduction estimates (mean: [Range]: (95 %CI))	overall observed streamflow reduction	long-term meteorological trend streamflow reduction estimates (mean: [Range]: (95 %CI))	overall observed streamflow reduction
1981–1995 versus 1996–2020 (pre- versus post-cap 1996)	17: [0–34.2]: (10.1–24.0)	17.1	11.5: [3.6–20.2]: (8.1–15.0)	38.9
1981–2000 versus 2001–2020 (baseline: equal split)	28: [16.5–39.7]: (23.1–33.0)	28	18.2: [12.1–25]: (15.7–20.8)	53
1981–2005 versus 2006–2020	1: [−0.7–2.9]: (0.3–1.7)	0.9	10.1: [8.4–11.8]: (9.4–10.8)	37.5

**Figure 7.**
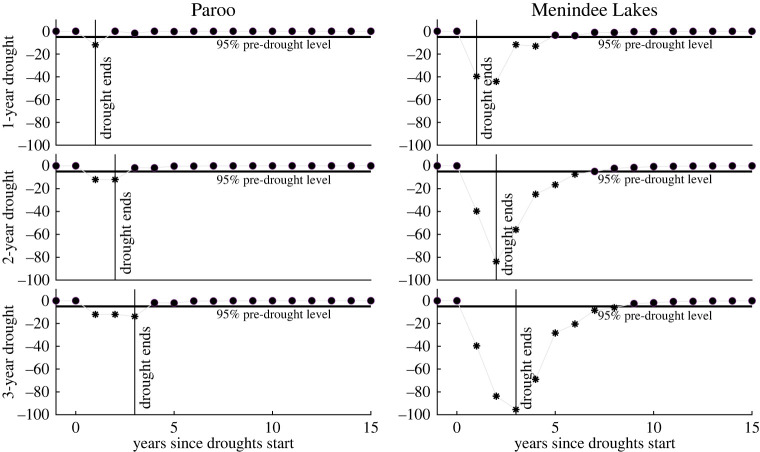
Modelled waterbird abundance responses to hydrological droughts. Responses are calculated as a percentage of the long-term median waterbird abundance level. Data points are plotted as asterisks when waterbird abundance has not recovered to 95% of its modelled pre-drought levels.

For these two time-length delineations (using a breakpoint year of 1996 and using a breakpoint year of 2006), estimates of the proportion of the decline in observed streamflow due to long-term meteorological trends were *lower*, and the proportion of the overall decline in streamflow attributable to anthropogenic drivers was *greater*, than when using the base-case breakpoint year of 2001. In sum, statistically significant declines in streamflow were not driven by changes of the breakpoint year. We also found, based on estimated shares of seasonal precipitation in annual precipitation, no statistically significant change in the seasonality of precipitation in either of the two catchments over the 40-year period (table 2).

**Table 2.** Changes in seasonal precipitation as percentage shares of annual precipitation, by catchments and trends between 1981–2000 and 2001–2020). Values were calculated by the authors from the SILO database. Estimates are rounded to two decimal places. Values inside parentheses are *p*-values rounded to the nearest two decimal places for the null hypothesis of a zero-time trend and *no* difference between the first 20-year average and the second 20-year average.

	Paroo River catchment		catchments of the Darling River	
	time trend over 40 years, 1981–2020 (%/year)	average difference for 2001–2020 versus 1981–2000 (%)	time trend over 40 years, 1981–2020 (%/year)	average difference for 2001–2020 versus 1981–2000 (%)
summer (Dec–Feb)	−0.14 (0.55)	−1.4 (0.79)	−0.1 (0.40)	−1.8 (0.50)
autumn (Mar–May)	−0.13 (0.44)	−0.64 (0.87)	−0.07 (0.51)	−1.1 (0.64)
winter (Jun–Aug)	0.1 (0.54)	0.53 (0.89)	0.1 (0.40)	1.8 (0.48)
spring (Sep–Nov)	0.17 (0.45)	1.5 (0.77)	0.07 (0.60)	1.1 (0.72)

### (d) Waterbird trends

Waterbird abundance declined in both wetlands, but the change differed by location (table 3). While the mean of waterbird counts in the 2001–2020 period declined by around 50% in the Paroo River Wetlands and adjacent areas, waterbird counts declined by about 75% at Menindee Lakes and adjacent areas compared to their level in the pre-2001 period. The coefficients of variation also declined proportionally more at Menindee Lakes.

**Table 3.** Descriptive statistics of waterbird abundance: Paroo River Wetlands and Menindee Lakes. Waterbird counts were available only from 1983 onwards.

		1983–2000	2001–2020
Paroo River Wetlands	mean (in 1000 counts) and range	37.36 [0.65–199.9]	15.81 [0–74.42]
	coefficient of variation	0.81	0.63
Menindee Lakes (adjacent to Barka River)	mean (in 1000 counts) and range	33.05 [2.1–144.5]	8.35 [0.01–50.86]
	coefficient of variation	0.84	0.56

Streamflow of the Paroo River and the Barka River had a statistically significant influence on waterbird abundance (table 4). This finding is consistent with previous evidence that streamflow is a key driver of waterbird populations [73,83,84]. The statistically significant association of streamflow and the lag of waterbird abundance with current waterbird abundance indicates that annual streamflow declines during a drought will negatively affect waterbird abundance. Further, this negative impact persists via the intertemporal connection of the lags. We further observed that the estimated coefficients of the lag of waterbird abundance in the Paroo River Wetlands are smaller than in Menindee Lakes, adjacent to the Barka River. Thus, when streamflow recovers to pre-drought levels, waterbird abundance in the Paroo River Wetlands and adjacent areas recovers more rapidly, implying greater resilience of waterbird abundance than at Menindee Lakes and adjacent areas.

**Table 4.** Summary of ADL estimates of waterbird abundance. Values inside parentheses are *p*-values rounded to three decimal places. Significance codes: * ≡ 0.1, ^**^ ≡ 0.05, ^***^ ≡ 0.01. Streamflow is summed within a water year defined by the months of October to September before the aerial waterbird surveys.

		Paroo River Wetlands	Menindee Lakes (adjacent to Lower Darling River)
intercept		4514	−2519
2nd lag of bird abundance		0.15 (0.102)	0.3^**^ (0.029)
local streamflow (as suggested by selection criteria)	current	25.47^***^ (<0.001)	5.48* (0.091)
	1st lag		6.1^**^ (0.042)
no. of observations		36	36
*R*^2^		0.36	0.42

We used the estimates in table 4 to simulate the impacts of hydrological drought-driven flow declines on waterbird abundance and evaluated how waterbirds might recover after a drought. For both regions and for different hydrological drought scenarios (figure 7), waterbird abundance declined but subsequently recovered following a hydrological drought. Waterbird abundance was more resilient at the Paroo River Wetlands than at Menindee Lakes, adjacent to the Barka River, in terms of both resistance and recovery time. Notably, the recovery time of waterbird abundance was longer at Menindee Lakes than at the Paroo River Wetlands because of the statistically significant impact of the lag of streamflow on waterbird abundance. The resistance of waterbird abundance to hydrological drought at Menindee Lakes, due to declines in streamflow, was also much less than at the Paroo River Wetlands.

### (e) Ecosystem resilience

**Figure 8.**
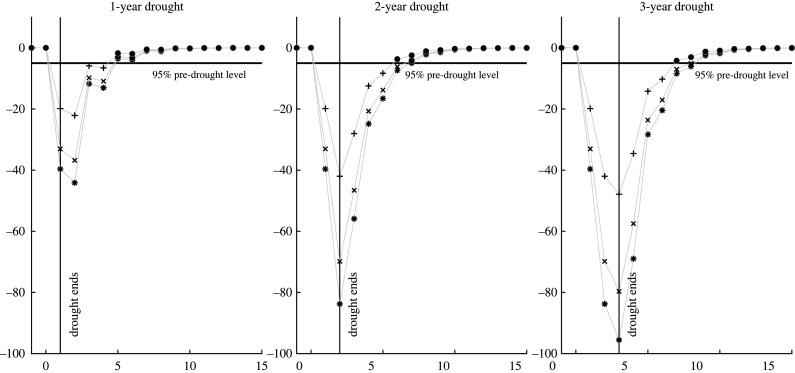
Estimated percentage changes in resilience (as measured by both resistance and recovery time) of waterbird abundance at Menindee Lakes with modelled water reallocations of 100 GL per year and 300 GL per year. Responses were calculated as a percentage of the long-term median waterbird abundance level. Data points for when waterbird abundance had *not* recovered to 95% of modelled pre-drought levels are plotted by asterisks under business as usual (no water reallocation), by crosses with a water reallocation of 100 GL per year to increase streamflow, and by plus signs with a water reallocation of 300 GL per year to increase streamflow.

The only feasible anthropogenic control over streamflow is to reduce upstream water extractions (and consumption). Figure 8 visualizes the estimated changes in waterbird abundance, our proxy for riparian ecosystem resilience, with two different levels of increased streamflow: an increase of 100 GL per year and an increase of 300 GL per year.

We assumed 80% irrigation efficiency [85] and estimated the costs of these two scenarios (100 GL and 300 GL per year increases) at approximately, per drought year, A$24 m (100 GL per year) and A$75 m (300 GL per year), or, respectively, around 1.3% (100 GL per year) and 4% (300 GL per year) of estimated total irrigation profits. We note that the reduction in irrigation is not proportional to the reduction in water reallocated from irrigation because the greater (smaller) the volume of water extracted, all else being equal, the lower (higher) the marginal net return from the last unit of water extracted.

The simulated results of the two mitigation options (100 GL per year and 300 GL per year), together with the business-as-usual case of no change in streamflow, showed that reduced upstream water extractions intended to increase downstream streamflow at Wilcannia (Barka River) promoted the resilience of waterbird abundance. Further, waterbird abundance recovered more quickly after the end of a hydrological drought with an in-stream water reallocation (100 GL or 300 GL per year). The greater the in-stream water reallocation to increase streamflow (300 GL versus 100 GL per year versus no increase), the more resilient was waterbird abundance to hydrological droughts (table 5). We found that waterbird abundance declined more and recovered less quickly during and after longer hydrological droughts, reduced upstream water extractions enhanced the resistance and recovery time of waterbird abundance, and improvements in resistance were substantial and more pronounced than recovery time under all three hydrological drought scenarios.

**Table 5.** Estimated resilience (resistance and recovery time) measures of waterbird abundance at Menindee Lakes.

	decline in waterbird abundance (%) [Resilience measure 1: *resistance*, defined as the percentage decline from pre-drought level]			approximate recovery time (to 95% pre-drought level) [resilience measure 2: *recovery time*, defined as the time-period, in years, to return to pre-drought level]		
hydrological drought length	1-year	2-year	3-year	1-year	2-year	3-year
no water reallocation	44	84	95	3.8	5.0	5.3
100 GL per drought year	37	70	80	3.7	4.6	5.0
300 GL per drought year	22	42	48	3.3	3.7	3.9

## 5. Discussion

We found evidence, based on meteorological time-series data, of a recent drying trend in the northern MDB, which is consistent with an observed drying trend in southern Australia ([86], p. 7). A warming climate, with more variable rainfall, may have increased actual evaporation [87], but actual evaporation is influenced by a number of factors including net radiation, wind speed, vapour pressure deficit and stomatal conductance reductions [88–90]. The observed meteorological drying trend has contributed to a decline in streamflow in the northern MDB and highlights that streamflow decline is also occurring in other arid and semi-arid environments [91].

### (a) Drivers of hydrological droughts

A key challenge when responding to hydrological droughts is to infer the effects of long-term meteorological trends on reduced streamflow from direct anthropogenic drivers such as changes in water extractions. The BoM and CSIRO [86] have observed declining streamflow in the Darling River region with half of these declining trends being statistically significant, and inferred that these streamflow declines are a result of climate change. The view that climate change is primarily responsible for reduced streamflow is also implied in the climate risk modelling undertaken by the NSW Department of Planning, Industry and the Environment [92], with its projections of reduced future precipitation. The MDBA in its ‘Working Scenario B, a Warmer and Drier Climate’ projects, by 2047–2075 relative to 1976–2005 [93], a decline in mean annual flow of 20–30% for the MDB [94]. This projected streamflow decline, because of climate change, is a smaller proportional decline in streamflow over the past 40 years for the Barka River caused by direct human influences alone.

Other, high-level assessments of human influences on reduced streamflow along the Barka River include the NSW Natural Resources Commissioner's review [95, p. 5]. The Commissioner observed: ‘Extractions following the commencement of the 2012 [Barwon–Darling] Plan rules have impacted significantly on baseflows, particularly downstream of Bourke [upstream of Wilcannia]. This has affected those communities and landholders reliant on the river for domestic and stock water supplies, town water supply, community and social needs’. The interim inspector-general of the Murray–Darling Basin [96, p. 12] reported that he was informed that the causes of declines in streamflow were water theft, lack of compliance, water extraction rules and floodplain harvesting. Further, the Australian Academy of Science’s report [38] concluded that the principal cause of the Barka River fish kills in 2019 was insufficient streamflow in the Barka River during hydrological droughts as a result of upstream extractions. In addition, the Wentworth Group of Concerned Scientists [97, p. iii] identified that expected streamflow along the Barka River between 2012–13 and 2018–19 fell by about one-third at two of the nearest upstream water gauges (Wilcannia and Louth) to Menindee Lakes. They attributed the possible causes to extractions of water intended for environmental purposes, lower than expected reliability of water rights held for the environment by governments, interception of overland flows via farm dams and floodplain harvesting, and changes in consumptive water use (such as the crops grown).

We contend that a key cause of the recent streamflow decline at Wilcannia is an increase in water extractions upstream of the Barka River. This is supported by quantitative evidence of large, unmetered and possibly increasing, water extractions associated with floodplain harvesting of the order of hundreds of GL per year in the northern MDB [23,38]. We also note a smaller estimate of increased metered ‘run-of-the-river’ water extractions, separate to floodplain harvesting, on the Darling River of 5–30 GL per year, over the period 2014–15 to 2017–18 relative to the period 1990–91 to 2012–13 [39, figs 3 and 4, p. 22].

### (b) Resilience to hydrological drought

Our findings indicated that streamflow declines have reduced waterbird abundance [73–75], which is strongly associated with breeding frequency when conditions are favourable [74,98]. Streamflow decline has, thus, affected the resilience (i.e. resistance and recovery time) of waterbird abundance. Of particular importance in relation to direct anthropogenic drivers of hydrological droughts, we found that the resilience of waterbird abundance is much greater in the Paroo River Wetlands and adjacent areas compared to Menindee Lakes and adjacent areas of the Barka River. This finding is consistent with observations from the Darling River [47] and elsewhere [99] that riparian ecosystems (and this is not only limited to waterbird abundance) that are less modified tend to be more resilient to adverse events, including hydrological droughts.

Some ecosystems may be able to withstand deteriorating conditions, within a given limit, and recover when environmental conditions improve. However, continued environmental deterioration can push ecosystems towards drier states from which recovery may not be straightforward [100,101]. We found that reduced resilience, as measured by the capacity of waterbirds to respond to favourable conditions, is already occurring at Menindee Lakes and associated wetlands. This finding is consistent with data on changes in mean trophic position and food chain length as a result of water resource development along the Barka–Darling River over the period 1869–2005 ([102], p. 6643).

Simulations of the reallocation of water from irrigation to increased streamflow during a hydrological drought indicated that the resilience of waterbird abundance can be improved by reducing upstream water extractions. Changes in the waterbird community, with its different feeding functional groups, can also represent broader changes in food resources, including invertebrates and vegetation in wetland ecosystems [103]. Increases in streamflow may also be supportive for other flora and fauna, not just waterbirds, such as fish, wetland plants and trees, mammals, frogs and reptiles [104]. Consequently, there may be additional co-benefits that we have not considered in our analysis of costs and benefits of water reallocation [105,106], noting that scheduling environmental water releases from dams to coincide with natural flood events may increase the resilience benefits of water reallocation [84].

**Figure 9.**
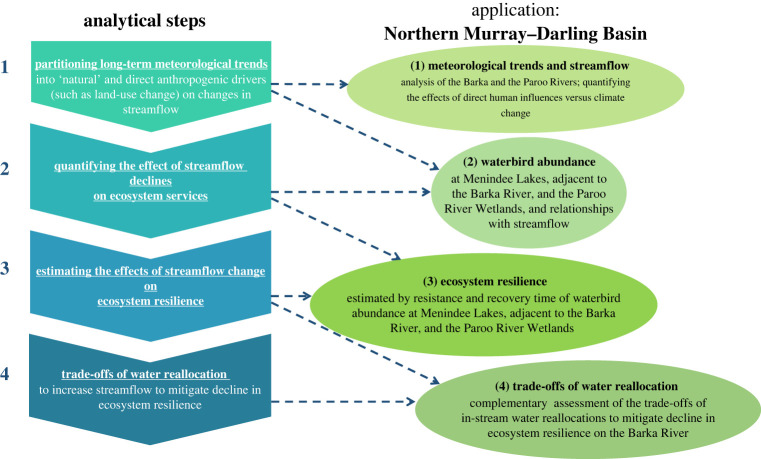
Four analytical steps in response to hydrological droughts and application to the Northern MDB. Source: the authors. (Online version in colour.)

Our four-step analyses in response to hydrological droughts in general, and specifically for the northern MDB, are summarized in figure 9. The four steps, in the context of the northern MDB, indicate two adaptive responses. First, improved publicly available measurements of extractions (especially in terms of floodplain harvesting), consumption, private water storages and return flows [107,108] would better partition the effects of long-term meteorological trends from direct anthropogenic drivers on streamflow decline. Second, water reallocations to increase streamflow, especially during hydrological droughts, would support riparian ecosystem resilience and, thus, help deliver on the key objects of the Water Act 2007 [109], as implemented in the 2012 MDB Plan and subsequent Basin Plans.

## 6. Conclusion

We developed a generally applicable four-step analysis framework that: (1) partitioned the causes of streamflow decline into long-term meteorological trends, possibly as a consequence of climate change and direct anthropogenic drivers; (2) estimated the consequences of streamflow decline on ecosystem services; (3) calculated the possible effects of streamflow decline, under different hydrological drought scenarios, on a measure of riparian ecosystem resilience; and (4) evaluated the costs and benefits of in-stream water reallocation to promote ecosystem resilience. Our approach was applied using meteorological, hydrological and ecological data in two catchments of the northern Murray–Darling Basin, Australia—a location that has features in common with other semi-arid catchments.

Step one of our analyses showed that over the past 40 years, direct anthropogenic drivers other than those associated with long-term meteorological trends have contributed to more than half of the recent decline in annual mean streamflow on the Barka River in the northern Murray–Darling Basin. In step two, we found that a decline in observed annual streamflow significantly and negatively affected waterbird abundance and waterbird resilience (resistance and recovery time) on both the Barka River and the Paroo River. Step three indicated that at Menindee Lakes, on the Barka River, (i) waterbird abundance is more sensitive to hydrological droughts, and (ii) waterbird abundance recovers more slowly than in the neighbouring Paroo River Wetlands, where there are virtually no water extractions. Step four estimated that the annual cost of water reallocation, intended to increase streamflow to increase a measure of riparian ecosystem resilience, represents only a small proportion of current irrigation benefits from upstream water extractions.

We contend that our four-step analytical framework is valuable for both understanding and adaptively responding to hydrological droughts. In particular, our framework partitions the impact of meteorological trends from regional-scale human influences, such as land-use change and water extractions. This partitioning is critically important if decision-makers are to quantify the causes of, and effectively respond to, streamflow decline. Our framework also assists decision-makers in assessing the impacts of streamflow decline on ecosystems and ecosystem resilience and evaluating the trade-offs of water reallocation. These four analytical steps, wherever there are accessible time-series data of sufficient quality, collectively support decision-making that promotes drought resilience.

## Data Availability

The datasets are available from the links provided in appendix A as well as from the authors .

## Appendix A

Data sources for figures and tables with GIS information and numerical values.

Figure 2

— Australia's state boundary: https://www.igismap.com/australia-shapefile-download/
— MDB boundary: https://data.gov.au/data/dataset/murray-darling-basin-boundary
— Major storage locations: http://www.bom.gov.au/waterdata/
— Indicative river lines: https://www.mdba.gov.au/sites/default/files/water-in-storages/weeklybasinreports/Basin-Storage-20220119.pdf
— Indicative locations of irrigation districts: https://www.mdba.gov.au/sites/default/files/pubs/1269-MDBA-Basin-map-poster-A1.pdf
— Location of Caiwarro station (#424201): http://www.bom.gov.au/waterdata/
— Location of Wilcannia station (#425008): https://realtimedata.waternsw.com.au/
— Location of waterbird survey: Richard Kingsford, Gilad Bino and Kingsford *et al.* [41]

Figure 4

— Temperature and precipitation in northern MDB (panels *a* and *b*): http://www.bom.gov.au/climate/data/acorn-sat/#tabs=Data-and-networks-
— Pan evaporation and dryness: data are calculated using the average of 1103 weather stations in the northern MDB; see https://www.longpaddock.qld.gov.au/silo/. The list of these weather stations is provided in the supplementary information section.

Figure 5

— Streamflow data at Caiwarro station (#424201): http://www.bom.gov.au/waterdata/
— Streamflow data at Wilcannia station (#425008): https://realtimedata.waternsw.com.au/

Figure 6

— Estimates of climatic impacts by MDBA [70]
— Observed reduction in streamflow: calculated from the sources of figure 4
— Baseline Budyko analysis results: estimated by the authors

Figure 7

— Modelled responses of waterbird abundance to hydrological droughts: estimated by the authors

Figure 8

— Estimated percentage changes in resilience of waterbird abundance at Menindee: estimated by the authors

Table 1

— Overall observed streamflow reduction in Caiwarro, Paroo River (station #424201): http://www.bom.gov.au/waterdata/
— Overall observed streamflow reduction in Wilcannia, Barka River (station #425008): https://realtimedata.waternsw.com.au/
— Other results: estimates by the authors

Table 2

— Statistical results are estimated by the authors from the data sources of table 1

Table 3

— Waterbird data are provided by Richard Kingsford and Gilad Bino

Table 4

— Econometric results are estimated by the authors

Table 5

— Resilience results are estimated by the authors

## Appendix B

Statistical analyses of changes in diversity, species richness and abundance between 1983–2000 and 2001–2020 are provided in table 6, and waterbird abundance and observed streamflow in tables 7–10.

**Table 6.** Test for changes in the Simpson diversity index (*SDI*), species richness and abundance of observed waterbirds between 1983–2000 and 2001–2020 at the Paroo River Wetlands and adjacent areas and at the Menindee Lakes and adjacent areas. We fail to reject the null hypothesis of no statistically significant change in *SDI* between the two periods in both locations at the 10% level of statistical significance. We reject the null hypothesis of no statistically significant change in species richness and abundance between the two periods and in both locations at the 10% level of significance. The *SDI* at location *r* in year *t* is calculated as $SDI_{r,t}=1-\sum_{S=1}^{S_{r}}\left(c_{r,s,t}/\sum_{S=1}^{S_{r}}c_{r,s,t}\right)$ where *S**_r_* is the total number of waterbird species recorded during the 1983–2020 period at location *r* and *c**_r,s,t_* is the abundance of species *s* at location *r* in year *t*. The *SDI* measures diversity on a scale between 0 and 1. If only one species were present in a year, the *SDI* in that year would be zero. If there were more than one species, the *SDI* would increase. The *SDI* approaches its upper bound of 1.0 when the proportional abundance of every individual species becomes smaller in the total population of waterbirds. Species richness is the number of identified waterbird species. Abundance is the count of all waterbirds.

		1983–2000 average	2001–2020 average	estimated change (*p*-value)
Paroo River Wetlands	*SDI*	0.73	0.70	−0.03 (0.57)
	species richness	28.83	17.45	−11.38 (<0.01)
	abundance (in 1000 counts)	37.36	15.81	−21.55 (0.08)
Menindee Lakes (adjacent to Barka River)	*SDI*	0.79	0.73	−0.06 (0.20)
	species richness	30.28	17.40	−12.88 (<0.01)
	abundance (in 1000 counts)	33.05	8.35	−24.71 (0.01)

**Table 7.** ADL specifications for the Paroo River Wetlands. Dependent variable is waterbird count at Paroo River Wetlands and adjacent areas. Values inside parentheses are standard errors. Significance codes: * ≡ 0.1, ^**^ ≡ 0.05, ^***^ ≡ 0.01. BIC = Bayesian information criterion.

intercept		8226.52	4141.85	7473.79	7892.23	4513.65
lag of bird abundance	first lag	−0.21 (0.18)	−0.26 (0.17)	−0.18 (0.14)	0.05 (0.09)	
	second lag	0.1 (0.11)	0.12 (0.11)	0.16* (0.09)		0.15 (0.09)
streamflow at Caiwarro (Paroo River)	current	21.83^***^	25.97^***^	26.25^***^	25.63^***^	25.47^***^
		(7.77)	(6.77)	(6.4)	(6.84)	(6.45)
	first lag	4.19 (9.08)	7.54 (7.83)			
	second lag	6.66 (8.81)	5.59 (7.69)			
streamflow at Wilcannia (Barka River)	current	−0.23 (3.39)				
	first lag	3.36 (3.46)				
	second lag	−4.14 (2.79)				
no. of observations		36	36	36	37	36
*R*^2^		0.47	0.42	0.39	0.3	0.36
BIC		840.53	833.19	827.57	852.83	825.88

**Table 8.** ADL specifications for the Lower Darling River region (Menindee Lakes and adjacent areas) Dependent variable is waterbird count at Menindee Lakes and adjacent areas. Values inside parentheses are standard errors. Significance codes: * ≡ 0.1, ** ≡ 0.05, *** ≡ 0.01 3. BIC = Bayesian information criterion.

intercept		2991.96	404.09	−1728.71	4569.17	−2519.23
lag of bird abundance	first lag	0.05 (0.17)	0.04 (0.16)	−0.07 (0.15)	−0.08 (0.14)	
	second lag	0.36^**^ (0.14)	0.37^**^ (0.13)	0.3^**^ (0.13)		0.3^**^ (0.13)
streamflow at Wilcannia (Barka River)	current	5.26 (4.02)	3.98 (3.26)	5.17 (3.26)	2.69 (2.91)	5.48* (3.14)
	first lag	9.08^**^ (3.92)	9.05^**^ (3.47)	6.71^**^ (3.24)	8.98^***^ (3.13)	6.1^**^ (2.89)
	second lag	−5.7 (3.63)	−5.43 (3.34)			
streamflow at Caiwarro (Paroo River)	current	−4.23 (9.1)				
	first lag	−5.29 (9.98)				
	second lag	1.48 (9.85)				
no. of observations		36	36	36	37	36
*R*^2^		0.48	0.47	0.43	0.34	0.42
BIC		852.76	842.62	842.08	866.15	838.71

**Table 9.** Poisson regression analysis for the Paroo River Wetlands. Dependent variable is waterbird count (10 000 counts) at Paroo River Wetlands and adjacent areas. Values inside parentheses are standard errors. Significance codes: * ≡ 0.1, ** ≡ 0.05, *** ≡ 0.01.

intercept		0.19	−0.03	0.09	0.13	−0.05
lag of bird abundance	first lag	−0.12 (0.07)	−0.10* (0.06)	−0.09 (0.05)	0.03 (0.03)	
	second lag	0.08^**^ (0.04)	0.07* (0.04)	0.08^***^ (0.03)		0.07^***^ (0.03)
local streamflow	streamflow	8.83^***^	8.24^***^	8.24^***^	8.37^***^	8.4^***^
		(2.36)	(1.85)	(1.61)	(1.62)	(1.61)
	first lag	−0.31 (3.19)	2.74 (2.72)			
	second lag	−3.41 (3.28)	1.04 (2.56)			
MDB streamflow	streamflow	−0.81 (3.03)				
	first lag	7.03^***^ (2.68)				
	second lag	−6.23* (3.34)				
no. of observations		36	36	36	37	36
BIC		154.09	155.52	149.41	162.32	148.72

**Table 10.** Poisson regression analysis for the Barka region (Menindee Lakes and adjacent areas). Dependent variable is waterbird count (10 000 counts) at Menindee Lakes and adjacent areas. Values inside parentheses are standard errors. Significance codes: * ≡ 0.1, ** ≡ 0.05, *** ≡ 0.01.

intercept		−2.31	−0.81	−0.9	−0.34	−0.84
lag of bird abundance	first lag	−0.05 (0.09)	0.05 (0.06)	0.03 (0.06)	−0.03 (0.05)	
	second lag	0.15^***^ (0.04)	0.15^***^ (0.04)	0.15^***^ (0.04)		0.14^***^ (0.04)
local streamflow	streamflow	−1.99 (2.4)	3.03^***^ (0.99)	3.30^***^ (0.95)	1.44^**^ (0.69)	3.1^***^ (0.85)
	first lag	3.38 (2.16)	2.47^***^ (0.93)	2.16^**^ (0.86)	3.64^***^ (0.77)	2.4^***^ (0.71)
	second lag	−3.6 (2.32)	−1.04 (1.11)			
MDB streamflow	streamflow	11.88^**^ (5.28)				
	first lag	0.25 (5.47)				
	second lag	8.47* (4.90)				
no. of observations		36	36	36	37	36
BIC		137.93	135.11	132.44	146.32	129.08
